# Trials by video link after the pandemic: the pros and cons of the expansion of virtual justice

**DOI:** 10.1007/s12689-022-00095-9

**Published:** 2022-04-13

**Authors:** D. L. F. de Vocht

**Affiliations:** Maastricht University, Maastricht, Netherlands

**Keywords:** Virtual justice, Remote justice, Technology and criminal procedure, Fair trial

## Abstract

The Covid-19 pandemic has led to an enormous increase in the use of technology in the courtroom. This development raises the important question on the potential effects of the digitalisation of criminal justice—especially from the viewpoint of the right to a fair trial. This contribution discusses this complicated question from different angles. It focuses on a number of different assumptions underlying the debate: the assumption that the use of technology in the courtroom diminishes human interaction, impedes an effective defence, influences decision-making and affects the legitimacy of the trial. This is done with the aim to shed light on the lack of evidentiary basis of these assumptions which clearly complicates the current discussion on the future of technology in the courtroom. The author argues that the validity of these assumptions needs to be adequately tested before we can make any long-term decisions on the content and scope of virtual criminal justice.

## Introduction

The COVID-19 pandemic has led to an enormous increase in the use of technology in the courtroom. By using video links and other alternatives to face-to-face communication, criminal justice systems all over the world have tried to reduce the number of people physically present in courtrooms as much as possible.^1^ This sudden rise of technology might create the impression that using technology for communication is a new phenomenon in the world of criminal justice. This is however not the case. Way before the pandemic many justice systems were already using technology to facilitate communication at a distance. It was—for example—already introduced in the United States as early as the seventies of the last century.^2^ Also in England and Wales, there had been a growing use of video link technology in first hearings and sentencing hearings already for many years.^3^ So gradually, more and more, defendants were appearing from courts and prisons via screens. In many systems these technological tools were also used for delivering expert evidence and hearing vulnerable witnesses. However, for many different reasons—such as lack of financial or technical means or traditional views on the importance of face-to-face communication in court—this pre-COVID development was rather slow and piecemeal. The pandemic radically changed this picture within a very short period of time. Criminal justice needed to continue its everyday work and closing the courts was not an option, at least not for long.

The fact that the COVID-19 crisis forced systems to adapt their practice and introduce digital means on a large scale within a short period of time, does not mean that technology will disappear from courthouses when COVID is ‘gone’. Without a doubt, technology is here to stay and it is fair to assume that the use of it will only increase further in the years to come. Even more so, it is realistic to assume that technology will expand in ways we cannot even begin to imagine today. When it comes to technology, the sky seems to be the limit.^4^ For this reason, it seems to be clear that the discussion on technology in the courtroom should not be about *yes or no (whether or not)*. After all, it is not the question *whether* technology will play a role in the criminal trials of—let’s say—2030: this is a given. The more relevant question is *to what extent and how* we want to allow it to replace physical face-to-face contact and adjust our traditional courtroom settings. In other words, what are the potential effects of the digitalisation of criminal justice—especially from the viewpoint of the right to a fair trial? Needless to say, this is a complicated question with many angles which cannot be dealt with extensively within the scope of this contribution. The aim of this article is to highlight some of the main assumptions that seem to dominate the discussion on digitalisation of criminal justice right now. In doing so I hope to shed some light on the lack of evidentiary basis of many of these assumptions which clearly complicates the current discussion. The focus will be on four different assumptions (which are by no means meant to be exhaustive): the assumption that the use of technology in the courtroom diminishes human interaction, impedes an effective defence, influences decision-making and affects the legitimacy of the trial.

As for definitions, it is important to note that many different concepts are used to describe the many different modalities of virtual justice. In this contribution the focus will be on so-called videoconferencing where some of the participants (mainly defendants and witnesses) participate from a remote location while others (judge, jury, public prosecutor) remain in the courtroom. The terms virtual justice, digital justice, remote justice and videoconferencing will be used as synonyms in this respect. Of course, the use of technology for communication during trial offers many more possibilities than ‘only’ videoconferencing with—for example—fully online trials being a realistic expectation for the (near) future.^5^

## Virtual justice diminishes human interaction

Traditionally, we tend to consider the administration of justice as a human business. This is especially true for criminal cases which often have a strong ‘human component’, with the eyes on the person of the defendant but (often) also on victims and witnesses. The result of this is that physical presence of all these participants is considered important as a prerequisite for delivering fair criminal justice.^6^ It allows the judge or jury to look the defendant in the eye and to see the witness when he or she gives a statement. We assume that communication—especially when delicate matters are being discussed—should ideally be done in a face-to-face setting. The increase of technology in the courtroom thus raises the important question: can justice be delivered without physical presence? Or will the use of technology result in losing the human element of justice, whatever that may be?

Needless to say, this question has many angles. For example: will seeing each other only on screen affect our human interaction? Will it affect the way we communicate (and most importantly understand each other)? Will participants to the proceedings lose the ability to take non-verbal cues of others into account? Does the use of technology create a barrier between the judge and the defendant and therefore result in a loss of—what some like to call—‘emotional connectivity’? These are only a few of the possible questions we face in this respect and we are nowhere near formulating any solid answers. Truth of the matter is we have no idea what the potential effects of socially distanced communication in a (criminal) justice context are. Main reason for this is that there is hardly any research available on this matter.

Despite the very limited amount of available research, there are clear indications that communication via screens is not the same as communication in person. For example, an older study conducted by Ferran and Wats illustrates that information processing is different when it is delivered via video conferencing in the sense that communication via screens has a heavier cognitive load than face to face communication.^7^ According to this theory, people in videoconferences tend to be more influenced by heuristic cues—such as how likeable they perceive the speaker to be—than by the quality of the arguments presented by the speaker. This as a result of the higher cognitive demands that this form of communication places on participants. As a result, people may focus more on form than on content or in other words: *what* is being said becomes less important than *how* it is said.

In addition to the fact that the way we perceive a message may be influenced by the way we communicate, there is also research illustrating that non-verbal communication and context information will be partially lost when trials are held via screens. This means that those participating to the proceedings will have to make a bigger effort to understand and ‘digest’ what is being communicated.^8^ Needless to say, any of these ‘extra’ difficulties accompanying remote communication will be especially challenging for vulnerable defendants such as defendants with mental disabilities. Although still rare, there are some pilot studies in the context of remote criminal justice which seem to confirm the difficulties mentioned above. It should however be mentioned that most of these studies are rather old (technology has evolved since then) and have been conducted within specific contexts (such as the use of video links in non-trial proceedings such as bail hearings in the United Kingdom and first appearances or arraignments in the United States). Nevertheless, they provide us with some useful insights. For example, a pilot study in the United Kingdom has illustrated that using video links might result in the fact that participants experience communication as more distant.^9^ Defendants indicated that they felt less ‘seen’ by the judge and have less opportunities to make a good impression.^10^ An evaluation study conducted by Plotnikoff and Woolfson illustrated that most interviewees (trial participants) felt that the use of video links did not have a positive effect on participation.^11^ More specifically, judges mentioned that they feared technology may have a freezing effect on defendants.^12^

The foregoing may give the impression that using technology will only have negative effects on the quality of communication. There is—however—also research indicating potential positive effects. For example, in the context of juveniles and young adults testifying via video link, there are studies illustrating that young witnesses feel less stress and experience less intimidation when they testify via video link.^13^ Also there are some pilots done in youth detention and correctional facilities in the United Kingdom and Australia where juveniles and young adults reported that they felt more familiar and more confident and at ease when using video link technology.^14^

These positive findings reported in the context of juveniles and young adults, give rise to another relevant question: is our conservative attitude towards technology in the courtroom maybe a generation ‘thing’ and will we eventually ‘grow out’ of it? Without a doubt, the virtual connection will feel more natural to those who grew up in the digital era. The children of today—who are the judges, the lawyers and also the defendants of tomorrow—have been using the internet and on-screen communication since birth. It is fair to assume that when technology becomes more and more integrated in our everyday lives, the feelings of unease that may go along with it, will slowly diminish. How fast we can adapt to new technological possibilities and realities has become very clear during the pandemic. Within no time, most people with ‘office jobs’ moved most of their work (including meetings) online. Many have experienced that online working and having meetings online can be a very efficient alternative to the traditional ‘day at the office’. Time will tell whether we will further grow into these new possibilities technology has to offer or that we will meet a cut-off point for accepting any more exceptions to face to face communication.

## Virtual justice impedes an effective defence

Very much linked to the question how technology affects communication, is the question on how it may influence an effective defence. As we all know, the European Convention on Human Rights (hereafter: ECHR) guarantees the right to effectively participate to the trial including the right to have a broad understanding of the nature of the process. One of the first questions that can be raised in this respect is whether the right to be present at trial—as enshrined in art. 6 ECHR—equals the right to be physically present. In this respect, it is important to note that the European Court of Human Rights (hereafter: ECtHR) has found that suspects or accused persons’ participation in proceedings by videoconference is not per se in conflict with the ECHR. However, the Court has also held that resorting to a video hearing is a restriction of the right to be present. Therefore, in any given case, the use of remote proceedings must serve a legitimate aim and the arrangements for giving evidence must comply with requirements for due process.^15^

We don’t know whether and to what extent appearing via video link will inhibit defendants to speak during trial. It is fair to assume that physical separation from the court may make it more difficult to actively participate in the proceedings but whether that is indeed the case: we don’t know. Does appearing on screen inhibit defendants to speak, ask for clarifications and interrupt the proceedings? Needless to say, it will very much depend on the capabilities, especially the communication skills of the defendant, whether he or she will be able to do so. In this respect, it is important to note that a substantial part of defendants suffer from some kind of mental disability or impairment. For many of these defendants, communicating via screens may be especially challenging. A telling example was given to me by a judge in the Limburg District Court who saw a female defendant with severe psychiatric problems via videoconferencing. The defendant could not understand the phenomenon of the court appearing on screen and was more or less excited that the ‘TV was talking to her’. This—of course—is just one example from a time where the pandemic forced us to deal with most cases remotely, but it clearly illustrates how delicate the matter can be when dealing with extra vulnerable defendants.

The use of technology and the lack of physical presence at trial may not only affect effective participation of the defendant himself but also the effectiveness of legal assistance. When the defendant appears on screen (and—for example—remains in the prison facilities) the lawyer will have to make a choice: either to go to prison and sit with his client or to go to court to be able to directly speak to the judge(s). In the Netherlands—during the pandemic—we have mainly seen examples of the latter: lawyers appearing in court waiving at their clients on screen. Needless to say, separating the lawyer from the defendant will undeniably impede the possibilities of confidential lawyer-client communication during trial. After all, not being in the same room will make it much more difficult to exchange information and clarify things during trial. The same is true for pre-and post-trial consultations: these will be much more difficult if not impossible when defendants are not physically in the courtroom. Also, it will be difficult to console or provide any type of emotional assistance to a defendant who appears via screen. While we know that this non legal aspect of providing legal assistance is valued as very important by many defendants.^16^ Pilots conducted in the United Kingdom have illustrated that both lawyers and defendants feel that remote justice has a negative impact on their relationship. Being separated from their lawyer could lead to the feeling that the latter lacks loyalty towards his client.^17^ When the lawyer chooses to be in the courtroom—a choice which is often made in the interest of an effective defence since it will make it easier to properly address the court—this may result in a feeling of ‘me against them’ for the defendant. Especially when, as for example sometimes happens in Dutch criminal proceedings—the lawyer is located close to the judge’s bench to make sure the defendant can see him or her on the screen. Although these appearances may be important, we should also not forget that some of these potential negative aspects of remote justice can be easily ‘corrected’ by making changes in how people are presented on screen (for example having separate ‘boxes’ for the different participants). Creating suitable practical solutions for any problems connected to confidentiality and the possibility to provide emotional support will most likely be more difficult.

Another aspect of effective defence that might be affected by the use of technology is the right to confront witnesses. Of course, this aspect of the right to defence is not equally relevant in all criminal justice system. It will—for example—be more pressing in the United Kingdom and Germany where hearing witnesses at trial is much more common than in for example the Netherlands where the use of written statements is the norm. This aspect of the right to defence will not be discussed further for now but it will definitely need to be taken into account when thinking about or discussing the future of technology in the criminal courtroom.

## Virtual justice may influence decision-making

Another relevant aspect of remote communication in courts, concerns the question whether (and if so—how) it may affect decision making. There are many ways in which the use of technology may affect the perception of decisionmakers (judge or jury) and their approach towards the defendant and his or her case. For example, as mentioned before, appearing on screen may have an impact on the likeability of the person appearing. Also, it may affect the decisionmaker’s view on the credibility, believability and voluntariness of his or her statements: an assumption which of course not only concerns the defendant but also other participants to the trial who give evidence such as witnesses and experts. More specifically, critics wonder what the effects are of defendants appearing from prison (possibly adding to a presumption of guilt?). Another relevant question is whether it is psychologically easier to decide in favour of the prosecution (to prolong detention, pass a guilty verdict, impose a higher sentence *et cetera*) when the decisionmaker does not meet the defendant in person. In other words: will a judge or jury be less sensitive to the negative impact of their decisions on the defendant when they only see him on a screen? Although the most fundamental question in this respect will of course be whether judgments of guilt are influenced by on screen appearances, it concerns not only the outcome of the trial but also the proceedings as such. For example, how does remote communication affect the possibility of the judge to assess whether a defendant is capable to stand trial? Also, will it not be more difficult for the judge to assess whether a defendant wants to ask a question, make a remark or needs additional explanation on a certain matter?

Although there is hardly enough available research to even begin to answer any of the questions mentioned above, the studies that *do* exist definitely suggest that the use of technology may influence the way defendants are perceived. For example, psychological research illustrates that defendants who appear on screen can come across as less believable and less authentic than those who appear in person.^18^ As mentioned before, there is also research indicating that appearing on video may affect your likeability. Therefore, judges may view defendants who appear by videoconference more negatively than those who physically appear in the courtroom.^19^ This assumption is supported by some pilot studies conducted in the United Kingdom and the United States where the use of videoconferencing led to a substantial increase in ‘negative decisionmaking’ (such as higher amounts of bail set and higher custodial sentences).^20^

As for the assessment of statements, there is research illustrating that testifying on screen may have a negative impact on believability. Some experimental studies show that recorded witness testimonies may be perceived as less reliable (by jurors) than ‘live’ testimonies.^21^ In an older study conducted by Goodman jurors reported that children who testify by closed circuit television rather than appearing live in court are less believable, less attractive, less intelligent, more likely to be making up a story, and less likely to be basing their testimony on fact versus fantasy.^22^ There is however also research indicating that statements made via video link are viewed as more reliable because the witnesses find themselves in a safer environment and—as a result—may experience less emotions and stress.^23^

Furthermore, we also know from existing research that certain practical aspects of technology—such as camera perspective—may be more influential than one might assume. See for example the work done by Lassiter and Irvine which shows that when a camera is only focused on a defendant during a videotaped interrogation, factfinders who view the videotape are more likely to believe that the defendant’s confession was voluntary and to find him guilty than if the camera focuses on the interrogator or on both the defendant and the interrogator.^24^ Also some of the effects on behaviour and perceptions may depend on how much of each participant is shown on the video screen and whether non-verbal communication such as gestures can be seen.^25^ This research illustrates that something as ‘trivial’ as the camera angle can in fact influence the perception of the judge and ultimately maybe also the outcome of the case.^26^

We should bear in mind that much of the aforementioned research concerns only witnesses (not defendants) and stems from times in which technology was definitely not as advanced as it is nowadays. We all know, technology moves and improves at an incredible pace meaning that the possibilities to transmit information (via sound and or image) become better every day.

## Virtual justice affects the legitimacy of the trial

One final assumption underlying the debate on potential effects of remote justice concerns the legitimacy of the trial. According to some, the use of technology and virtual attendance of key participants such as the defendants will negatively affect the so-called ‘solemnity of the trial’. According to this line of reasoning, the court is more than just a location: it is a symbolic and maybe even ‘sacred’ place with ritualistic functions. A trial—with all its traditions—is held in a physical courtroom for a reason: it underlines the importance of what is being discussed and the values at stake. As expressed by Rowden and Wallace who specifically refer to the role and image of the judge in court: “The judge embodies the authority of the court, as an adjudicator and as the authority responsible for managing the court and the other courtroom participants. Any dissonance between the image of the judge and the nature of their role potentially detracts from their effectiveness because courts, unlike other branches of government, essentially rely on public acceptance of their legitimacy”.^27^ The question of course is whether virtual presence will negatively affect these characteristics, and—if so—whether that is necessarily a bad thing. Are we holding on to traditions without reason or will remote participation indeed affect the legitimacy of the trial?

One of the more specific concerns in this respect is that defendants appearing on screen may have a diminished sense of the seriousness of the proceedings.^28^ Will the defendant—who appears before the court from prison or maybe even from his own home—still realise what is at stake? The meeting with the court is an important encounter in the sense that it symbolizes the settlement of a ‘conflict’ with the state. Having a day in court is important for the perception of the defendant in more than one way. Not only to have him or her realise the importance of what is going on (giving the state the opportunity to make him aware of the wrongdoing he or she is being accused of) but also to illustrate to the defendant that his or her case is dealt with in a transparent and legitimate way. In the words of the European Criminal Bar Association: “*Criminal justice is also about participants “having their day in court”, that is, having their side of the story heard and understood. The right of the accused to look the court in the eye in a public hearing, while making his or her statement or pleadings, is a corollary of the fair trial and of human dignity.”*^29^

In addition to this, remote participation raises the question what the court can do when a defendant behaves ‘badly’ on screen. A defendant who is physically present in court, is under ‘control’ of the presiding judge in the sense that he or she can be disciplined and—if necessary—even removed from the courtroom in case of misconduct. Needless to say, the court loses this control when the defendant appears on a screen. In case of defendants appearing from prison, the court will have to rely on prison staff to deal with unwanted behaviour. In a way, video conferencing means that a third-party (such as prison authorities in charge of setting up the video link) gains control over—at least part of—the trial. Furthermore, one could also argue that experiencing the ‘official’ atmosphere of the courtroom is important in the context of truth telling in the sense that being physically present may encourage those who are testifying to speak the truth.

Although the questions and reservations mentioned above are certainly relevant and valid, they are also speculative and lacking evidentiary basis. When studying and discussing the possible effects of technology on the legitimacy of the trial in the future we should not forget that—in time—it may be possible to re-enact some of the courtroom surroundings that now often get lost in virtual settings. The possibilities of virtual and augmented reality seem to be almost limitless so creating an online interactive experience of a real-world trial may not be as far-fetched as it sounds. Only time will tell whether technology will be able to imitate the physical trial to a satisfactory degree and—even if we can—whether we want to ‘settle for’ the virtual version. In this respect we should also not forget the advantages the use of technology may have for the publicity (openness) of the trial. Although some fear that technology creates barriers to open justice (for example in Dutch trials nowadays, screens in some courts are only visible for the court and not for the public meaning that the latter can only hear but not see the defendant appearing on screen), it may also potentially increase publicity. As we know, criminal trials in most Western legal systems are—as a rule—open for the public but the number of visitors is generally low. Only in high profile cases which get a lot of media attention this will be different. This means that we often feel that the court system and judicial practice is detached from society. Maybe the use of technology could improve this by making it easier for people to follow trials—for example online via live streams as already happens in some cases of international tribunals. If the larger public has a general understanding of what goes on in court this could potentially strengthen the legitimacy of trials and criminal justice in general.

## Some final remarks

As I have tried to illustrate, there are many assumptions about how digitalisation may affect trials, their participants and the process of decision-making. In a nutshell, these assumptions seem to fuel the idea that the positives of remote justice are (only) on the side of the state: using technology makes proceedings cheaper and faster and—as such—it is often presented as a cost-effective solution to the widespread problem of overloaded and in many ways dysfunctional justice systems. However, this is not as straightforward as it may seem. We simply don’t know whether the potential advantages are only on the side of the state because we don’t know whether the assumptions described above—mostly based on the idea that the quality of the trial, the effective participation of the defence, the process of decision making and maintaining judicial authority will be negatively affected by the use of technology—are in fact true. Is it really the case that the (perceived) benefits are only on the governmental side of the system and not on the side of the defendant or—more generally—the fairness of proceedings? Both sides of the debate—those in favour on the one hand and those opposed on the other—use arguments based on certain assumptions on the effects of technology in the courtroom but none of these arguments seems to be evidence based.

As I have tried to illustrate there is a huge lack of research in this respect. The research that *is* available offers valuable insights but is often outdated and conducted in specific (sometimes not even criminal justice) contexts. We know technology offers us huge potential but without a clear evidentiary basis for deciding which way to go with technology in the courtroom, we don’t know how to use this potential the best way possible. More specifically, we need to test the validity of the assumptions described above, before we let them decide how we proceed.

The timing for more in-depth research seems to be right. The experiences justice systems all over the world have had with remote participation during the pandemic offer a wealth of information. We can—and should—definitely learn from these experiences but we should also not forget that the COVID-19 situation was (and at present still is) an extraordinary one. It was a global emergency situation calling for fast and far going measures. We should take this into account to avoid that the impact COVID-19 has (had) on criminal justice blurs the discussion on how to proceed with digitalisation in the courtroom. More in-depth research can provide us with the necessary insight and knowledge and help us to deepen the discussion. It will allow us to transcend the current level of the debate based mainly on assumptions and decide whether and to what extent the values and principles we want to protect are actually affected by the use of technology in the courtroom. Only then will we be able to make informed decisions on making remote participation a permanent feature of our justice systems. Much work is still to be done.

## Footnotes

1 For an overview of worldwide developments in this respect and national reports on how criminal justice systems have been affected by the COVID19 outbreak, see the national reports available at the website of Fair Trials International: https://www.fairtrials.org/covid19justice.

2 The first use of video technology in the legal system occurred in an Illinois court in 1972 to conduct a video bail hearing: Davis et al (2015).

3 On the possible effects of remote participation on the quality of justice in the United Kingdom: Gibbs (2017).

4 In the words of Susskind: “No-one in the world of tech seems to be saying ‘enough is enough’. Enough, it seems, is not nearly enough. In fact, the pace of technological change itself is accelerating. This follows from the scale of current investment in technology, both capital and brain power (…) it seems to me much more likely that by say, 2030, our daily lives (and our courts) will have transformed by what I call ‘as-yet-uninvented’ technologies”, (Susskind 2019, p. 10).

5 In the United Kingdom the NGO Justice has been testing the concept of fully remote jury trials, where all participants join the virtual court via video, with the hearing livestreamed to a virtual public gallery. See Justice’s website for the different evaluations of these mock trials: https://justice.org.uk/our-work/justice-covid-19-response/.

6 As eloquently voiced by A. Langdon (QC) during his inaugural speech as Chairman of the Bar of England and Wales (2016): “The humanity of physical presence is, I suggest, an important component in the delivery of justice… Being in the physical presence of a witness or a jury or a defendant or a judge or your lawyers… isn’t that fundamental to our innate sense of how justice should be delivered? … Justice has a human face, and it’s not a face on a screen… Many smaller cases benefit from getting everyone together in one place. The dynamic between the parties becomes evident; whether one side is unfairly dominating the other, whether one party is as well-heeled as the other”, mentioned in Susskind (2019, p. 206).

7 See Ferran and Watts (2008/2009, p. 1569).

8 Bohannon et al (2013, pp. 177–185).

9 Fielding et al (2020, pp. 97–98).

10 Fielding et al (2020, pp. 107).

11 Plotnikoff and Woolfson (2000, p. 28 and further).

12 Plotnikoff and Woolfson (2000, p. 28–30 and 36–37).

13 Goodman et al (1998, p. 165).

14 McKay (2018) and Walsh (2018, pp. 61–181).

15 See for example the cases Marcello v. Italy (ECtHR 5 October 2006, nr. 45,106/04) and Sakhnovskiy v. Russia (ECtHR 2 November 2010, no. 21272/03). It should be mentioned however that these cases concerned situations in which remote hearings were held due to specific circumstances of the case (such as security issues).

16 See for example: Kemp (2010, p. 84).

17 Fielding et al (2020, p. 66).

18 See for a summary of relevant studies: Bowen Poulin (2004 pp. 1087–1167).

19 Treadway Johnson and Wiggins (2006, p. 222) referring to research conducted by Bengtsson and others (n.d.) who found that participants who worked with a partner on a decision-making task rated partners with whom they interacted in person more sociable, likeable, dynamic, and truthful than partners whose input was presented via a computer.

20 Fielding et al (2020*,* p. 104) and Seidman Diamond et al (2010, pp. 869–902, pp. 877–878 and p. 897).

21 Landström (2007, pp. 344–45), Goodman et al (1998, pp. 165–203 and pp. 195–196) and Orcutt et al (2001, pp. 339–372, pp. 357–358 and p. 366).

22 Goodman et al (1998, p. 199).

23 See for example: Ellison and Munro (2014, pp. 3–29).

24 Lassiter et al (1986). See also more recent on the existence of a so-called camera perspective bias: Lassiter et al (2009, pp. 157–170).

25 Treadway Johnson and Wiggins (2006, p. 222).

26 We should not forget – however – that these experimental studies concern witnesses and not defendants so their relevance for the topic at hand should not be overestimated.

27 Rowden and Wallace (2018, p. 504).

28 Treadway Johnson and Wiggins (2006, p. 215).

29 European Criminal Bar Association, Statement of Principles on the use of Video-Conferencing in Criminal Cases in a Post-Covid-19 World, September 6, 2020, available at: https://www.ecba.org/extdocserv/20200906_ECBAStatement_videolink.pdf, pp. 14–15, where it is also mentioned: “A trial in a court is not a random physical and geographical setting, but an essential requirement of fair and human criminal justice. Punishment imposed by video dehumanised the accused and weakens the effect of the trial procedure in restoring legal peace. A trial taking place in person allows the confrontation between the accused and the victims or witnesses to publicly demonstrate the innocence of the acquitted person, and to make the guilty person aware of his or her wrongdoing and the evidence to demonstrate it, thereby also reducing further re-offending. Remote trials are simply not capable of producing the same effect”.
